# Bisphosphonates and Cancer: What Opportunities from Nanotechnology?

**DOI:** 10.1155/2013/637976

**Published:** 2013-03-04

**Authors:** Giuseppe De Rosa, Gabriella Misso, Giuseppina Salzano, Michele Caraglia

**Affiliations:** ^1^Department of Pharmacy, Università degli Studi di Napoli Federico II, Via Domenico Montesano 49, 8013 Naples, Italy; ^2^Department of Biochemistry, Biophysics and General Pathology, Seconda Università degli Studi di Napoli, Via Costantinopoli 16, 80138 Naples, Italy

## Abstract

Bisphosphonates (BPs) are synthetic analogues of naturally occurring pyrophosphate compounds. They are used in clinical practice to inhibit bone resorption in bone metastases, osteoporosis, and Paget's disease. BPs induce apoptosis because they can be metabolically incorporated into nonhydrolyzable analogues of adenosine triphosphate. In addition, the nitrogen-containing BPs (N-BPs), second-generation BPs, act by inhibiting farnesyl diphosphate (FPP) synthase, a key enzyme of the mevalonate pathway. These molecules are able to induce apoptosis of a number of cancer cells *in vitro*. Moreover, antiangiogenic effect of BPs has also been reported. However, despite these promising properties, BPs rapidly accumulate into the bone, thus hampering their use to treat extraskeletal tumors. Nanotechnologies can represent an opportunity to limit BP accumulation into the bone, thus increasing drug level in extraskeletal sites of the body. Thus, nanocarriers encapsulating BPs can be used to target macrophages, to reduce angiogenesis, and to directly kill cancer cell. Moreover, nanocarriers can be conjugated with BPs to specifically deliver anticancer agent to bone tumors. This paper describes, in the first part, the state-of-art on the BPs, and, in the following part, the main studies in which nanotechnologies have been proposed to investigate new indications for BPs in cancer therapy.

## 1. The Bisphosphonates

**Figure 1:**
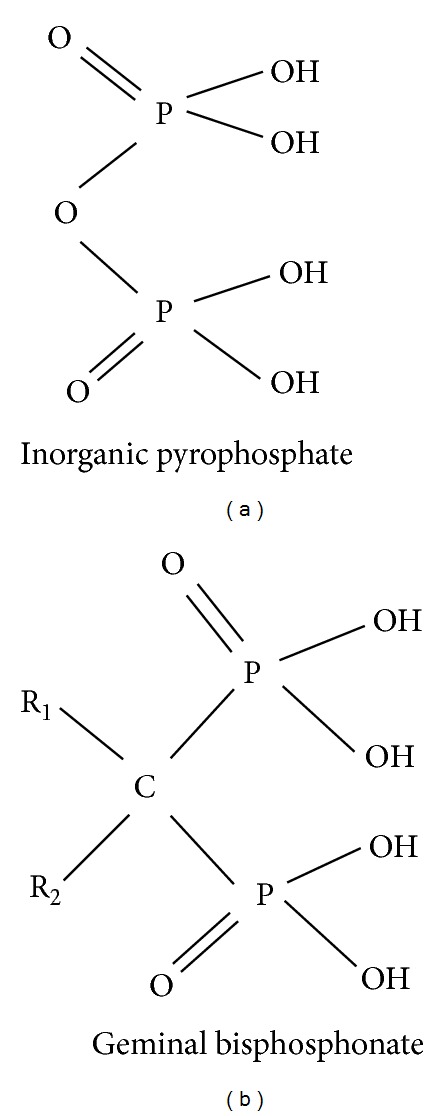
Structures (a) and (b) show the basic structures of inorganic pyrophosphate and geminal bisphosphonate, respectively, where R_1_ and R_2_ represent different side chains for each bisphosphonate.

Bisphosphonates (BPs), synthetic analogues of naturally occurring pyrophosphate compounds, represent the treatment of choice for different diseases, such as metabolic bone disease, osteoporosis, Paget's disease, and bone metastases [1]. In the 1960s Fleisch et al. proposed that inorganic pyrophosphate, a naturally occurring polyphosphate and a known product of many biosynthetic reactions in the body, might be the body's own natural “water softener” that normally prevents calcification of soft tissues and regulates bone mineralization by binding to newly forming crystals of hydroxyapatite [2, 3]. It subsequently became clear that calcification disorders might be linked to disturbances in inorganic pyrophosphate (PPi) metabolism [2, 3]. Alkaline phosphatase present in bone destroys pyrophosphate locally, thereby allowing amorphous phase calcium phosphate to crystallize and inducing mineralization of bone [2]. The major limitation of pyrophosphate is that, when orally administered, it is inactive because of its hydrolysis in the gastrointestinal tract. During the search for more stable analogues of pyrophosphate that might also have the antimineralization properties of pyrophosphate but would be resistant to hydrolysis, several different chemical classes were studied. The bisphosphonates (at that time called diphosphonates), characterized by P–C–P motifs, were among these classes [1–4]. The fundamental property of BPs, which has been exploited by industry and medicine, is their ability to form bonds with crystal surfaces and to form complexes with cations in solution or at a solid-liquid interface. Since BPs are synthetic analogues of pyrophosphates, they have the same chemical activity, but greater stability [1–4]. Like pyrophosphates, BPs had high affinity for bone mineral and they were found to prevent calcification both *in vitro* and *in vivo* but, unlike pyrophosphate, they were also able to prevent experimentally induced pathologic calcification when given orally to rats *in vivo*. This property of being active orally was key to their subsequent use in humans [4]. Perhaps the most important step toward the successful use of BPs occurred when their ability to inhibit hydroxyapatite crystals dissolution was demonstrated. This finding led to following studies designed to determine if they might also inhibit bone resorption [5]. The clarification of this property made BPs the most widely used and effective antiresorptive agents for the treatment of diseases in which there was an increase in the number or activity of osteoclasts, including tumor-associated osteolysis and hypercalcemia [6]. After more than three decades of research, first-, second-, and third-generation bisphosphonates have been developed. Changes in chemical structure have resulted in increased potency, without demineralization of bone [1]. There is now a growing body of evidence regarding the efficacy of these drugs in clinical settings. All BPs that act significantly on the skeleton are characterized, as stated above, by P–C–P bond (Figure 1(a)), in contrast to pyrophosphate, which has a P–O–P bond (Figure 1(b)).

**Figure 2:**
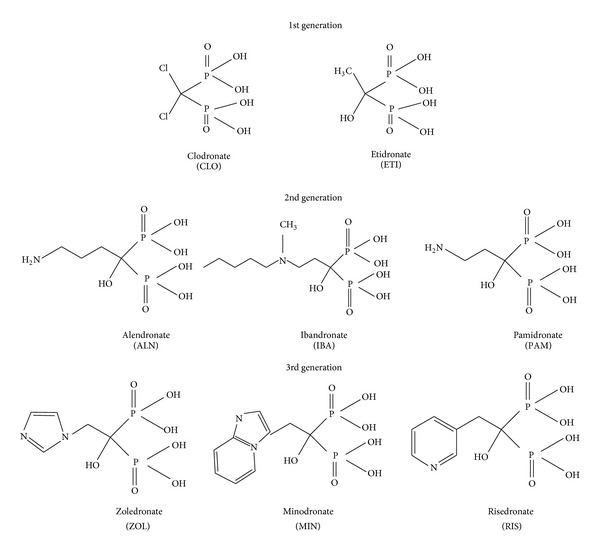
Structures of simple bisphosphonates (1st generation), N-BPs with primary, secondary, or tertiary nitrogen function in the R_2_ alkyl side chain (2nd generation) and N-BPs with heterocyclic rings in the R_2_ side chain (3rd generation).

This peculiarity confers stability both to heat and to most chemical reagents and is one of the most important properties of these compounds [4]. Extensive chemical research programs have produced a wide range of molecules with various substituents attached to the carbon atom. Variations in potency and in the ability of the compounds to bind to crystals in bone one determined by the chemical and three-dimensional structure of the two side chains, R_1_ and R_2_, attached to the central, geminal carbon atom [1–4]. The bioactive moiety comprising the R_2_ chain of the molecule is considered primarily responsible for BPs' effect on resorption, and small changes in this part of the structure can result in large differences in their antiresorptive potencies [4]. The uptake and binding to bone mineral is determined by the bi- or tridentate ligand (hydroxybisphosphonate) of the molecule, which is also thought to be responsible for the physicochemical effects, the most important being the inhibition of growth of calcium crystals. The most effective structures for binding to bone mineral consist of the two phosphonate groups attached to the central carbon and the substitution at R_1_ with a hydroxyl or amino group that provides tridentate binding [4]. In fact, the addition of a hydroxyl (OH) or primary amino (NH_2_) group increases the affinity for calcium ions, resulting in preferential localization of these drugs to sites of bone remodelling. Increasing the number of carbon atoms in the side chain initially increases and then decreases the magnitude of the effect on bone resorption [1–4]. The early compounds, clodronate (CLO) and etidronate (ETI), contained simple substituents (H, OH, Cl, CH_3_) and lacked a nitrogen atom (Figure 2). 

Subsequently, more complex and potent compounds were produced by the insertion of a primary, secondary, or tertiary nitrogen function in the R_2_ side chain, for example, pamidronate (PAM), alendronate (ALN), ibandronate (IBA), and incadronate (INC), which have an alkyl R_2_ side chain, or risedronate (RIS), zoledronate (ZOL), and minodronate (MIN), which have heterocyclic rings in the R_2_ side chain (Figure 2). Variation of the substituents modulates the pharmacologic properties and gives each molecule its unique profile [7].

## 2. Intracellular Effect and Pharmacodynamics of Bisphosphonates 

Extensive structure/activity studies have resulted in several very useful drugs that combine potent inhibition of osteoclastic bone resorption with good clinical tolerability [5–8]. The pronounced selectivity of BPs for bone rather than other tissues is the basis for their value in clinical practice. The antiresorptive effect cannot be accounted simply by adsorption of BPs to bone mineral and prevention of hydroxyapatite dissolution. It became clear that BPs must inhibit bone resorption by cellular effects on osteoclasts rather than simply by physicochemical mechanisms [5]. Bisphosphonate moiety and R_1_ group are both essential for hydroxyapatite affinity [8]. The BPs bind to hydroxyapatite crystals in the area of osteoclast-mediated bone erosion; during resorption, the dissolution of hydroxyapatite crystals by osteoclast determines the consequent release of the bisphosphonate that may indeed come into contact with osteoclasts and inhibit their absorption capacity [8]. Incorporation of an aminoalkyl side chain at R_2_ increases antiresorptive potency by 10-fold; also, the length of carbon chain is important (alendronate is about 1000-fold more potent than etidronate while pamidronate is only 100-fold more active than etidronate) [4, 8]. In addition, incorporation of a nitrogen heterocycle (third-generation agents) further enhances antiresorptive potency: the most active compound in this class is ZOL, a BP containing an imidazole ring, which is up to 10000-fold more potent than both CLO and ETI in some experimental systems. During bone resorption, BPs are probably internalized by endocytosis along with other products of resorption [4, 8]. Many studies have shown that BPs can affect osteoclast-mediated bone resorption in a variety of ways, including effects on osteoclast recruitment, differentiation, and resorptive activity, and may induce apoptosis [7]. Because mature, multinucleated osteoclasts are formed by the fusion of mononuclear precursors of hematopoietic origin, BPs could also inhibit bone resorption by preventing osteoclast formation, in addition to affecting mature osteoclasts. *In vitro*, BPs can inhibit dose-dependently the formation of osteoclast-like cells in long-term cultures of human bone marrow [7]. In organ culture, also, some BPs can inhibit the generation of mature osteoclasts, possibly by preventing the fusion of osteoclast precursors [5]. In contrast to their ability to induce apoptosis in osteoclasts, which contributes to the inhibition of resorptive activity, some experimental studies suggest that BPs may protect osteocytes and osteoblasts from apoptosis induced by glucocorticoids [9].

Since the early 1990s there has been a systematic effort to elucidate the molecular mechanisms of action of BPs, and, not surprisingly, it has been found that they could be divided into 2 structural subgroups [10, 11]. The first group comprises the nonnitrogen-containing bisphosphonates, such as CLO and ETI, that perhaps most closely resemble pyrophosphate. These can be metabolically incorporated into nonhydrolyzable analogues of adenosine triphosphate (ATP) methylene-containing (AppCp) nucleotides, by reversing the reactions of aminoacyl-transfer RNA synthetases [12]. The resulting metabolites contain the P–C–P moiety in place of the *β*,*γ*-phosphate groups of ATP [13]. Intracellular accumulation of these metabolites within osteoclasts inhibits their function and may cause osteoclast cell death, most likely by inhibiting ATP-dependent enzymes, such as the adenine nucleotide translocase, a component of the mitochondrial permeability transition pore [14]. Induction of osteoclast apoptosis seems to be the primary mechanism by which the simple BPs inhibit bone resorption, since the ability of CLO and ETI to inhibit resorption *in vitro *can be overcome when osteoclast apoptosis is prevented using a caspase inhibitor [15].

**Figure 3:**
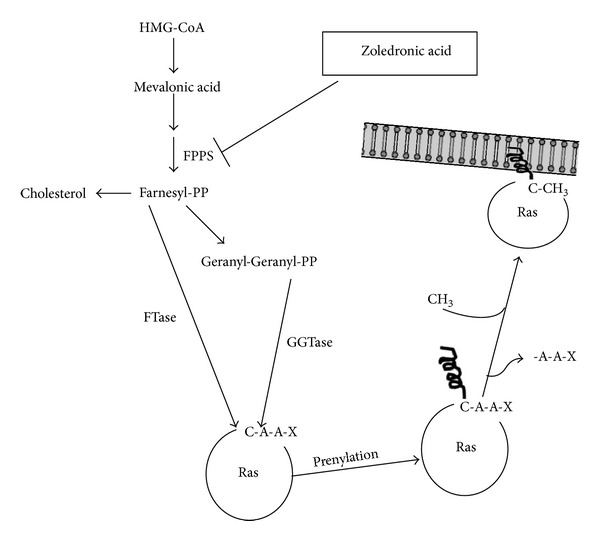
Isoprenoids are synthesized from the mevalonate pathway that starts from reaction catalyzed by the 3-hydroxy-3-methylglutaryl CoA (HMG-CoA) reductase (the rate-limiting reaction in cholesterol biosynthesis) which catalyzes the conversion of HMG-CoA to mevalonic acid. The pathway triggered by this reaction can lead to the synthesis of a key isoprenoid molecule, the farnesyl-pyrophosphate (Farnesyl-PP), whose formation is catalyzed by the farnesylpyrophosphate synthase (FPPS). Farnesyl-PP can be either converted by a series of reactions in cholesterol or can be transferred on target cellular proteins as Farnesyl-PP itself (reaction catalyzed by farnesyltransferase, FTase) or firstly converted in geranyl-geranyl-pyrophosphate (Geranyl-Geranyl-PP) and then transferred on cellular proteins by type I or type II geranylgeranyl-transferase (GGTase). FTase and GGTase-I catalyze the prenylation of substrates with a carboxy-terminal tetrapeptide sequence called a CAAX box, where C refers to cysteine, A refers to an aliphatic residue, and X typically refers to methionine, serine, alanine, or glutamine for FTase or to leucine for GGTase-I. Following prenylation of physiological substrates, the terminal three residues (AAX) are subsequently removed by a CAAX endoprotease, and the carboxyl group of the terminal cysteine is methyl esterified by a methyltransferase. At this moment prenyl substrates, such as Ras, are ready to be located on the inner side of the biological membranes to receive signals mediated by external factors. ZOL specifically inhibits the FPPS activity required for the synthesis of farnesyl and geranylgeranyl lipidic residues blocking prenylation of Ras that regulates the proliferation, invasive properties, and proangiogenic activity of human tumour cells.

In contrast, the second group, comprising the nitrogen-containing bisphosphonates (N-BPs), which are several orders of magnitude more potent at inhibiting bone resorption *in vivo* than the simple bisphosphonates, is not metabolized to toxic analogues of ATP [16]. N-BPs act by inhibiting farnesyl diphosphate (FPP) synthase, a key enzyme of the mevalonate pathway (Figure 3).

This enzyme is inhibited by nanomolar concentrations of N-BPs. ZOL and the structurally similar MIN are extremely potent inhibitors of FPP synthase [6] and inhibit the enzyme even at picomolar concentrations. Importantly, studies with recombinant human FPP synthase revealed that minor modifications to the structure and conformation of the R_2_ side chain that are known to affect antiresorptive potency also affect the ability to inhibit FPP synthase. These studies strongly suggest that FPP synthase is the major pharmacologic target of N-BPs in osteoclasts *in vivo* and help to explain the relationship between bisphosphonate structure and antiresorptive potency [6]. Clinical and experimental evidence indicates that N-BPs suppress the progression of bone metastases, and recent observations suggest that this effect may be independent of the inhibition of bone resorption [17]. Tumour progression and metastasis formation are critically dependent on tumour angiogenesis [18]. Antiangiogenic treatments suppress tumour progression in animal models, and many antiangiogenic substances are currently being tested in clinical trials for their therapeutic efficacy against human cancer [19]. Recent research indicates that ZOL possesses antiangiogenic activities [20]. 

The exact mechanism by which N-BPs inhibit FPP synthase is only just becoming clear. The recent generation of X-ray crystal structures of the human FPP synthase enzyme, cocrystallized with RIS or ZOL [51], revealed that N-BPs bind the geranyl diphosphate (GPP) binding site of the enzyme, with stabilizing interactions occurring between the nitrogen moiety of the N-BP and a conserved threonine and lysine residue in the enzyme. Enzyme kinetic analysis with human FPP synthase indicates that the interaction with N-BPs is highly complex and characteristic of “slow tight binding” inhibition [51]. By inhibiting FPP synthase, N-BPs prevent the synthesis of FPP and its downstream metabolite geranylgeranyl diphosphate [11]. These isoprenoid lipids are the building blocks for the production of a variety of metabolites, such as dolichol and ubiquinone, but are also required for posttranslational modification (prenylation) of proteins, including small GTPases [11]. The loss of synthesis of FPP and geranylgeranyl diphosphate therefore prevents the prenylation at a cysteine residue in characteristic C-terminal motifs of small GTPases, such as Ras, Rab, Rho, and Rac (Figure 3). Small GTPases are important signaling proteins that regulate a variety of cell processes important for osteoclast function, including cell morphology, cytoskeletal arrangement, membrane ruffling, trafficking of vesicles, and apoptosis. Prenylation is required for the correct function of these proteins because the lipid prenyl group serves to anchor the proteins in cell membranes and may also participate in protein-protein interactions [3, 20].

## 3. Pharmacokinetics of Bisphosphonates

Recent studies with a fluorescently labelled bisphosphonate have shown that macrophages and osteoclasts internalize bisphosphonates into membrane-bound vesicles by fluid-phase endocytosis; endosomal acidification then seems to be absolutely required for exit of bisphosphonate from vesicles and entry into the cytosol [52]. This mechanism of uptake suggests that large amounts of N-BP is in intracellular vesicles but probably only very small amounts of bisphosphonate then enter in the cytosol or in other organelles for inhibition of FPP synthase. Even though, the relatively poor uptake of bisphosphonates into the cytosol is overcome by their extremely potent inhibition of FPP synthase [6, 11]. Bisphosphonates are poorly absorbed in the intestine due to their negative charge hindering their transport across the lipophilic cell membrane; they are therefore given mainly intravenously. A pharmacokinetic evaluation of ZOL for treatment of multiple myeloma and bone metastases, carried out by Ibrahim et al., exhibited a three-compartment model [53]. The distribution half-life (*α*-*t*_1/2_) was 14 min, followed by a *β*-phase of 1.9 h. A prolonged terminal phase, with a half-life of at least 146 h, might indicate a slow release of ZOL from the bone back into the plasma. ZOL pharmacokinetics were dose proportional from 2 to 16 mg based on peak plasma concentration (*C*_max⁡_) and area under the curve (AUC_24 h_). ZOL dosed every 21 days did not demonstrate significant plasma accumulation. *In vitro *studies indicated that 22% of ZOL is protein bound. The excretion of ZOL was primarily renal. Approximately 40% of the radiolabeled ZOL dose was recovered in urine within 24 h. Only traces of ZOL were observed in the urine after two days, suggesting a prolonged period of ZOL binding to bone. Population modeling described the ZOL clearance as a function of creatinine clearance. On the basis of a comparison of AUC_24 h_, patients with mild or moderate renal impairment had 15 and 43% higher exposure, respectively, than patients with normal renal function. However, no significant relationship between ZOL exposure (AUC) and adverse events might be established. The use of ZOL in patients with severe renal failure was not recommended. *In vitro *studies showed no inhibition of or metabolism by cytochrome P-450 enzymes [53]. 

One of the most important limits of N-BPs, which makes the direct anticancer activity difficult to demonstrate *in vivo*, is just their pharmacokinetic profile. This issue is demonstrated by also other pharmacological studies performed on different N-BPs. In fact, after intravenous administration (4 mg over 15 min) of ZOL, an immediate increase of its concentration in peripheral blood was recorded, as shown by estimations of the early distribution and elimination of the drug, which resulted in plasma half-lives of the drug of about 15 min (*t*_1/2*α*_) and of 105 min (*t*_1/2*β*_), respectively. The maximum plasma concentration (*C*_max⁡_) of ZOL was about 1 *μ*M, that was from 10- to 100-fold less than that required in *in vitro* studies to induce apoptosis and growth inhibition in tumour cell lines, while the concentrations required for anti-invasive effects were in the range of those achieved after *in vivo* administration. Moreover, approximately 55% of the initially administered dose of the drug was retained in the skeleton and was slowly released back into circulation, resulting in a terminal elimination half-life (*t*_1/2*γ*_) of about 7 days [54, 55]. Other studies performed on ALN demonstrate that N-BP concentration in noncalcified tissues declined rapidly at 1 h (5% of the initial concentration). On the other hand, its concentration in the bone continuously increased, reaching its peak at 1 h, demonstrating that a significant redistribution of the drug from noncalcified tissues to bone occurred. The drug was retained in bone tissue for a long time and was slowly released into plasma, with a terminal half-life of about 200 days [56]. Similar data were obtained with IBA and ZOL [54–57] demonstrating that long-lasting accumulation in bone is a common feature of N-BPs. The rapid redistribution of N-BPs results not only in a short exposure of noncalcified tissues to the drug, but also in a prolonged accumulation in bone where N-BPs can also reach higher and tumoricidal concentrations. These considerations explain the relative efficacy of N-BPs on tumours placed in bone tissues [20]. In biodistribution studies by Weiss et al. performed in rats and dogs administered with single or multiple intravenous doses of ^14^C-labeled ZOL, its levels rapidly decreased in plasma and noncalcified tissue, but higher levels persisted in bone and slowly diminished with a half-life of approximately 240 days. In contrast, the terminal half-lives (50 to 200 days) were similar in bone and noncalcified tissues, consistent with ZOL rapidly but reversibly binding to bone, being rapidly cleared from the plasma, and then slowly released from bone surfaces back into circulation over a longer time. The results suggested that a fraction of ZOL is reversibly taken up by the skeleton, the elimination of drug is mainly by renal excretion, and the disposition in blood and noncalcified tissue is governed by extensive uptake into and slow release from bone [58]. It is important to consider that ZOL is not taken up by tumor cells but prevalently by cells with increased endocytosis processes such as osteoclasts and macrophages. However, owing to the intrinsic pharmacokinetics limitations of ZOL, more efforts were required to increase the anticancer activity of both this drug and the other members of N-BPs family.

## 4. Bisphosphonate and Cancer: *In Vitro* Studies

FPP synthase is a highly conserved, ubiquitous enzyme; therefore, N-BPs have the potential to affect any cell type *in vitro*. Among BPs recent advances suggest that ZOL, beyond the strongest activity of antibone resorption, has direct anticancer effects. In fact, extensive *in vitro* preclinical studies support that ZOL can inhibit tumor cell adhesion to extracellular matrix proteins, thereby impairing the process of tumour-cell invasion and metastasis [59]; moreover, it was demonstrated that ZOL has a direct effect on angiogenesis *in vitro* [60, 61] and an *in vitro* stimulation of *γ*/*δ* T lymphocytes, which play important roles in innate immunity against cancer [62]. One of the crucial mechanisms responsible for the antitumor activity of ZOL is the induction of tumor cell apoptosis [63].

Inhibition of protein prenylation by N-BPs can be shown by measuring the incorporation of ^14^C mevalonate into farnesylated and geranylgeranylated proteins [64]. The most potent FPP synthase inhibitor, ZOL, almost completely inhibits protein prenylation in J774 cells at a concentration of 10 *μ*mol/L, which is similar to the concentration that affects osteoclast viability *in vitro *[65]. Alternatively, the inhibitory effect of N-BPs on the mevalonate pathway can be shown by detecting accumulation of the unprenylated form of the small GTPase Rap1A, which acts as a surrogate marker for inhibition of FPP synthase and which accumulates in cells exposed to N-BPs. Roelofs et al. have shown the ability of N-BPs to inhibit the prenylation of Rap1A in a wide range of cultures of different types of primary cells and cell lines such as osteoclasts, osteoblasts, macrophages, epithelial, and endothelial cells, and breast, myeloma, and prostate tumor cells [16]. Macrophages and osteoclasts were the most sensitive to low concentrations of N-BPs (1–10 *μ*M) *in vitro*. Moreover, treatment with 100 *μ*M N-BP caused a detectable accumulation of unprenylated Rap1A already after few hours. Concerning myeloma cells, in order to detect the unprenylated form of Rap1A, longer times of *in vitro* treatments and higher concentrations were required [16].

BPs have also been shown to inhibit adhesion of tumor cells to extracellular matrix (ECM) proteins and to promote invasion and metastasis. Inhibition of the mevalonate pathway and induction of caspase activity are important mechanisms in explaining the inhibitory effects of N-BPs on tumor cells adhesion to the ECM and on invasiveness [66]. *In vitro* findings have demonstrated that N-BPs, particularly ZOL, can affect endothelial cells exerting a suppressive effect on angiogenesis [67, 68]. In fact, N-BPs inhibit the expression of vascular endothelial growth factor (VEGF) and platelet-derived growth factor (PDGF) that induce the proliferation of endothelial cells and enhance the formation of capillary-like tubes.

Recent evidence suggests that ZOL is a potent inducer of apoptosis in several cancer cell types [69]. It has recently been demonstrated *in vitro* that N-BPs, PAM and ZOL, induce apoptosis and growth inhibition in human epidermoid cancer cells, together with depression of Ras-dependent Erk and Akt survival pathways. These effects occurred together with poly(ADP-ribose) polymerase (PARP) fragmentation and the activation of caspase 3 [70]. Moreover, the latter seems to be essential for apoptosis induced by N-BPs in this experimental model. Furthermore, it was reported that ZOL induced growth inhibition on both androgen-dependent LnCaP and androgen-independent PC3 prostate cancer cell lines with G1 accumulation. Recent studies showed that the effects of ZOL were caspase dependent. In human breast cancer cell lines, ZOL induced a modulating expression of Bcl-2 and subsequent caspase 3 activation. These events might be precipitated by inhibition of Ras activation, which requires protein farnesylation [71].

In human colon carcinoma HCT-116 cells, ZOL strongly inhibited the proliferation paralleled by a G1 cell cycle accumulation and induction of apoptosis *via *a caspase-dependent mechanism [72]. Recent studies by Fujita et al. demonstrated the involvement of the mevalonate pathway in the antiproliferative and proapoptotic effects of ZOL on ACHN renal cell carcinoma cells [73]. 

The sensitivity of different cell types to N-BPs most likely depends largely on their ability to internalize sufficient amounts of N-BPs to inhibit FPP synthase. In view of the pharmacokinetic concerns that limit the anticancer activity of ZOL, in the last decade the scientists have defined a series of pharmacological and molecular strategies. Some approach was represented by the design of rationale-based drug combinations and the improvement of the pharmacokinetic profile. Evidence from both *in vitro *and *in vivo *models indicated a synergistic antitumor activity of N-BPs when used in combination with either cytotoxic drugs or targeted molecular therapies [69]. Based on the relevance of the farnesylation inhibitory effects on antitumour activity of N-BPs, the farnesyl transferase inhibitor (FTI) R115777 was used together with PAM or ZOL, and the effects of the combination treatment on growth inhibition and apoptosis were evaluated. N-BPs and FTI given in combination were strongly synergistic [70]. Notably, low concentrations of FTI induced a strong increase of Ras expression with only a moderate reduction of Ras activity that was, on the other hand, significantly reduced by the combined treatment [70]. These data suggested that escape mechanisms for the inhibition of isoprenylation of Ras might be based on the geranylgeranylation or other prenylating processes [74]. The addition of farnesol to cells treated with the combination abolished the effects of the N-BPs/FTI combination on apoptosis and on the activity of the signaling molecules, suggesting that the synergistic growth-inhibitory and proapoptotic effects produced by the N-BPs/FTI combination involved the inhibition of both Erk and Akt survival pathways acting in these cells in a Ras-dependent fashion [70].

 A synergistic interaction between R115777 and ZOL was also found on both androgen-independent PC3 and androgen-dependent LNCaP prostate cancer cell lines [70], and the effects were attributed to enhanced apoptosis and inactivation of Erk and Akt. Several papers reported the significant cytostatic and cytotoxic effects of docetaxel (DTX) and ZOL on the hormone- sensitive prostate cancer cell line, LNCaP [17, 75, 76]. In details, the highest inhibition of cell proliferation was observed after DTX exposure and was already evident at concentrations 200-fold lower than the plasma peak level. Fabbri et al. hypothesized the use of low DTX doses in concomitance with and followed by a prolonged ZOL exposure to reduce the prostatic tumour cell population and to rapidly induce eradication of hormone-resistant cells present in hormone-responsive tumours, without compromising the use of conventional-dose DTX for the first-line treatment for hormone-sensitive prostate cancer. The principal molecular mechanisms involved were found to be apoptosis and decreased pMEK and Mcl-1 expression [77]. Furthermore, Karabulut et al. found that the combination treatment of DTX and ZOL in hormone and drug refractory, PC-3 and DU-145 prostate cancer cells, synergistically inhibited cell growth by inducing the apoptotic pathways through the downregulation of the antiapoptotic protein Bcl-2 [78]. 

A further strategy for the implementation of ZOL activity is the interference of its molecular targets. The recent analysis—performed by cDNA microarray platform—of gene modulation induced by ZOL in androgen-resistant prostate PC3 cell line showed a significant dose- and time-dependent reduction of transcriptional activity of CYR61 after exposure to ZOL, as demonstrated by the reduction of the transcriptional activity of Cyr61 promoter [79]. This result is considered of interest in designing new therapeutical approaches in androgen-independent prostate cancer.

## 5. Bisphosphonate and Cancer: *In Vivo* Studies

In addition to the established *in vitro* induction of tumor cell apoptosis, also emerging *in vivo* evidence supports N-BPs anticancer activity. Preclinical studies support that ZOL displays an antitumor activity, including direct antitumor *in vivo* effects such as inhibition of tumor cell adhesion to mineralized bone, invasion and effects on angiogenesis (animal models) probably due to the modification of various angiogenic properties of endothelial cells [59–61]; effects on the metastatic process (animal models) [60]; stimulation of *γ*/*δ* T lymphocytes in humans [62]. N-BPs may target several steps involved in the metastatic process, extracellular matrix, extravasation into distant tissues, angiogenesis, and avoidance of immune surveillance [80].

Roelofs et al. detected the unprenylated form of Rap1A in osteoclasts purified from ALN-treated rabbits using immunomagnetic beads, thereby showing that N-BPs inhibit protein prenylation *in vivo *[16].

Many animal studies have focused on models of multiple myeloma, breast cancer, and prostate cancer showing that the newer N-BPs can significantly reduce the number and size of osteolytic lesions in tumor-bearing mice, reduce skeletal tumor burden, induce tumor cell apoptosis in bone lesions, reduce serum levels of tumor markers, and prevent formation of bone metastases [81–83]. 

A recent study, utilizing a plasmacytoma xenograft model without complicating skeletal lesions, demonstrated that treatment with ZOL led to significant prolongation of survival in severe combined immunodeficiency mice inoculated with human INA-6 plasma cells. Following treatment with ZOL, histological analysis of tumors revealed extensive areas of apoptosis associated with poly(ADP-ribose) polymerase cleavage. Furthermore, western blot analysis of tumor homogenates demonstrated the accumulation of unprenylated Rap1A, indicative of the uptake of ZOL by nonskeletal tumors and inhibition of farnesyl pyrophosphate synthase [84]. This is one of the few evidence of direct antitumor effects of N-BPs in plasma cell tumors *in vivo. *In fact, it is generally believed that the reduction in tumor burden observed in some animal models may be due to inhibition of osteoclast activity [85]. For example, bisphosphonates, including IBA and ZOL acid, were shown to inhibit the development of osteolytic bone lesions in the 5T2MM model and alternative models of myeloma bone disease [86]. Moreover, the effect of bisphosphonates on the osteoclast stimulatory activity (OSA) was evaluated in the marrow of patients with multiple myeloma. For this purpose, the effects of IBA treatment prior to the development of bone disease were examined in a murine model of human myeloma. Sublethally irradiated severe combined immunodeficient (SCID) mice were transplanted with ARH-77 cells on day 0. These ARH-77 mice were treated daily with subcutaneous injections of N-BP started before or at different times after tumor injection. ARH-77 mice were sacrificed after they developed paraplegia, and the data demonstrated that early treatment of ARH-77 mice with IBA prior to development of myeloma bone disease decreases OSA and possibly retards the development of  lytic lesions but not eventual tumor burden [87]. Numerous studies in breast cancer models have also been reported. A study using MDA-MB-231 human breast tumour cells injected directly into the femoral artery of male athymic rats also showed that IBA (10 *μ*g/kg/day, days 18 to 30) reduced the extent of the osteolytic lesions [88]. This study also provided evidence that once tumours have reached a certain size (>6 mm in this model) they become less dependent on the bone microenvironment for their further expansion, and hence less sensitive to BP therapy. A study by van der Pluijm and colleagues showed that BPs modify tumour growth primarily through effects on bone, rather than through targeting tumour cells directly [89]. MDA-231-B/luc+ breast cancer cells were implanted by intracardiac injection, and olpadronate given as a preventive (subcutaneous 1.6 *μ*mol/kg/day from 2 days before implantation) or a treatment (days 3 to 43) schedule. Effects on the formation of new bone metastases and osteolysis were assessed, as well as tumour burden, both inside and outside the bone marrow cavity. However, the reduction in tumour growth was only transient and did not affect progression of established tumours. Studies in a prostate cancer model have also recently been reported. In those studies PC-3 and LuCaP cells were injected directly into the tibia of mice [81], PC-3 cells form osteolytic lesions, and LuCaP cells form osteoblastic lesions. The treatment group receiving ZOL (5 *μ*g s.c. twice weekly) either at the time of tumor cell injection or after tibial tumors was established (7 days for PC-3 tumors and 33 days for LuCaP tumors). Treatment with ZOL significantly inhibited growth of both osteolytic and osteoblastic metastases by radiographic analysis and also reduced skeletal tumor burden, as evidenced by a significant decrease in serum levels of prostate-specific antigen in animals bearing LuCaP tumors. The observed reduction in serum prostate-specific antigen levels provides compelling direct evidence of the antitumor activity of ZOL in this animal model. The potential of ZOL to prevent bone metastasis was also demonstrated in an animal model of prostate cancer [90].

In order to separate the direct antitumour effects of BPs from those mediated via bone, the sequential or combined treatment with other antitumor agents were investigated. 

The synergistic interaction between R115777 and ZOL on both androgen-independent PC3 and androgen-dependent LNCaP prostate cancer cell lines was also found to induce cooperative effects *in vivo *on tumour growth inhibition of prostate cancer xenografts in nude mice with a significant survival increase [70]. These *in vivo *and *in vitro* effects were in both cases attributed to enhanced apoptosis and inactivation of Erk and Akt.

On the basis of preliminary results about sequence-dependent synergistic effects of ZOL and DTX combination on growth inhibition and apoptosis of human prostate cancer cells, the closely related taxane, paclitaxel (PTX), has shown synergistic inhibitory activity with ZOL in animal models for lung cancer. Compared with vehicle and ZOL alone, cancerous cells in the bone of mice treated with PTX + ZOL expressed higher levels of Bax and lower levels of Bcl-2 and Bcl-xl. Moreover, this drug combination produced a significant reduction in serum n-telopeptide of type I collagen which levels correlate with the rate of bone resorption. The results of this study indicated that ZOL enhanced the efficacy of PTX synergistically, by reducing the incidence of bone metastasis from lung cancer and prolonging survival in a mouse model of nonsmall cell lung cancer with a high potential for metastasis to bone [91]. 

Ottewell et al. also showed that the treatment with ZOL after exposure to doxorubicin (DOX) elicited substantial antitumor effects in a mouse model of breast cancer. Interestingly, the treatment induced an increase in the number of caspase-3-positive cells paralleled by a decrease in the number of tumour cells positive for the proliferation marker Ki-67. Moreover, the sequential treatment with clinically relevant doses of DOX, followed by ZOL, reduced intraosseous but not extraosseous growth of breast tumours in mice injected with a clone of MDA-MB-231 [92].

The findings of synergy of interaction between ZOL and other agents could reduce the ZOL concentrations required for antitumour activity and then could allow the achievement of its effective *in vivo *levels, overcoming the limits associated with the pharmacokinetics of ZOL.

Another strategy to potentiate the antitumor effects of chemotherapeutic agents and ZOL could be also the administration of the drugs at repeated low doses (“metronomic” way). Santini et al. recently demonstrated that weekly administration of ZOL has higher antitumor effects as compared with conventional 3 weekly administration in nude mice xenografted with breast cancer cells, even if the total administered dose is the same [93]. Moreover, a single dose of 1 mg ZOL is able to induce a significant reduction of circulating VEGF in patients with bone metastases suggesting an *in vivo* biological activity of low ZOL concentrations in humans [93].

## 6. Nanotechnology and BPs: Macrophage Targeting

Macrophages are the major differentiating cell of the mononuclear phagocyte system (MPS). They derive from monocytes that migrate from the peripheral blood to extravascular tissue where they differentiate into macrophages [94]. Macrophages play a critical role in host defense because they migrated to an infected focus following attraction by a variety of substances, such as components from bacteria, complement components, immune complexes, and collagen fragments. Once at the infected focus, macrophages may phagocytose and kill infectious agents by a variety of mechanisms [95]. Moreover, following uptake of protein antigens, macrophages generated immunogenic fragments activating and regulating the immune response [96]. Finally, macrophages infiltrate tumors, where they represent an important mechanism of host defense against tumor cells, either inhibiting tumor cell division or killing the cells following secretion of soluble mediators or by other means [97, 98]. However, most tumors can be infiltrated by a different macrophage phenotype, which provides an immunosuppressive microenvironment for tumor growth. Furthermore, these tumor-associated macrophages (TAM) secrete many cytokines, chemokines, and proteases, which promote tumor angiogenesis, growth, metastasis, and immunosuppression [99]. 

Thus, due to their pivotal role in a number of physiological and pathological processes including tumors, macrophages represent an attractive target for therapy. While in the case of small soluble drug, only a small fraction can reach the macrophages, these latter can be the preferential accumulation site for intravenously injected colloidal carriers. Indeed, once into the bloodstream plasma proteins adsorb on particle surface and this process, also named opsonization, facilitates particle recognition and clearance from the blood by circulating phagocytes as well as tissue macrophages that are in direct contact with the blood [100]. Thus, the localization of intravenously injected nanocarriers in cells of the mononuclear phagocytes system (MPS) offers a potential and powerful method to target therapeutic agents to these cells. Nowadays, various lipid and polymeric carriers such as liposomes and nanoparticle are under investigation to deliver drugs to macrophages. However, nanocarrier characteristics, in terms of size, shape, and particle surface, affect the pharmacokinetics of the nanocarrier and need to be carefully evaluated when designing nanocarriers for macrophage targeting. For more details, the readers are directed to more specific reviews on this theme, for example, an excellent review by Moghimi [100].

The powerful effect of BPs against osteoclasts suggests a possible activity on cells with a common lineage, such as the macrophages. However, pharmacokinetics of BPs require delivery method to escape bone and to target macrophages. Liposomes encapsulating CLO were successfully used to achieve temporary macrophage depletion in the spleen [21]. The authors demonstrated that once phagocytosed, the liposomal membranes were disrupted by the phospholipases of the lysosomes, and the drug is released into the cell. Other studies confirmed macrophage elimination from the spleen, following intravenous (i.v.) injection of CLO entrapped into liposome by the absence of lysosomal acid phosphatase activity [21, 22] and surface markers of macrophages [23] as well as by the absence of cells with the capacity to ingest and accumulate carbon particles from the circulation [22]. Ultrastructural studies also confirmed that macrophages not only lose some of their functional characteristics but are also physically removed from the circulation [26]. Growth inhibition of macrophages-like cells by using liposomes encapsulating BP was also confirmed with other BPs, namely, PAM and ETI, on RAW 264 and CV1 cells [24]. In this study, free BPs were found to be even 1000 times less active, compared with the corresponding liposome-based formulations. Interestingly, the use of high calcium extracellular concentration resulted in a stronger macrophage depletion, suggesting the role of calcium to mediate BP cell uptake [24, 27]. The liposome type affected macrophage depletion, which was higher when using negatively charged unilamellar liposomes [27]; however, this effect was found only in the case of CLO and ETI but not in the case of PAM. Finally, the use of calcium/bisphosphonate complex was found to lead to an enhanced uptake into cells but not to an inhibitory effect on the cytokine production by macrophages [27]. BP-encapsulating liposomes, when intravenously administered, led to elimination of macrophages from spleen and liver [25] but not those in other organs [23], reflecting the pharmacokinetics of the carrier. Accordingly, subcutaneous footpad administration of the BP-encapsulating liposomes resulted in macrophage elimination in draining lymph nodes [28] while intratracheal administration exclusively eliminates macrophages from lung tissues [29].

Liposome encapsulating BPs were used to enhance tumor growth in an experimental model of liver metastasis [30]. Rat inoculation with colon carcinoma cells resulted in a strong enhanced tumor growth in the liver only when the animals were pretreated with an i.v. injection of CLO-encapsulating liposomes. This effect was attributed to the effective elimination of all Kupffer cells that are preferential accumulation site for colloidal carriers. Accordingly, in the same experiment, nonphagocytic cells into the liver were not affected [30]. In contrast, liposome encapsulating CLO have been successfully used to inhibit the tumor growth. In different experimental animal models of cancer, this effect was accompanied by drastic reduction of the blood vessel density in the tumor tissue [31–33, 101]. CLO-encapsulating liposomes were also used in combination therapy with VEGF-neutralizing antibody. The treatment led to significant reduction of angiogenesis, as demonstrated by blood vessel staining and vessel quantification, that was associated to a significant reduction of the TAM and tumor-associated dendritic cells [31]. Liposomes encapsulating CLO were also investigated in combination with sorafenib in two human hepatocellular carcinoma xenograft nude mouse models [34]. Mice treated with sorafenib showed a significant inhibition of tumor growth and lung metastasis but associated to significant increase of macrophage recruitment in peripheral blood as well as increased intratumoral infiltration. A combination therapy with sorafenib and liposome containing CLO or sorafenib and free ZOL also led to reduced tumor angiogenesis, with the highest effects found with ZOL. This effect could be surprising when considering that zoledronic acid was used as free; however, the strong activity of ZOL at very low concentrations, compared with CLO, could explain the highest effect found in this study. In the same study, the authors found toxic effects in animals treated with liposomes encapsulating CLO, while ZOL appeared as more promising, especially because already in the clinical practice. Macrophage depletion by using BP-containing liposomes has also been proposed as adjuvant agent in the cancer radiotherapy. Indeed, radiotherapy, although directly inducing tumor cell death, may upregulate proangiogenic and prosurvival factors within the tumor microenvironment. In particular, upon radiation, upregulation of tumor cells and cells of the myeloid lineage can occur, with consequent TNF*α* production [35] followed by the induction of macrophage-secreted vascular endothelial growth factor (VEGF) with consequent radioprotective effect. Radiotherapy association with the treatment with CLO-containing liposomes resulted in the improvements in the therapeutic index, as determined by a delay of tumor regrowth [36]. The use of CLO-containing liposomes was also useful to reduce metastasis of human prostate cancer in bone, thus confirming the role of TAM in regulation of tumor tissue homeostasis [37]. The effect was potentiated when mice were inoculated with cancer cells, previously knocked down of IL-6, thus confirming the role of IL-6 as a strong chemotactic factor that recruits TAM to the tumor lesion.

## 7. Nanotechnology and BPs: Targeting of Cancer Cells 

Although many research papers are focused on the use of nanocarriers targeting macrophages, the delivery of bisphosphonates directly to cancer cells has been recently investigated. 

Tumors characterized by cells derived from myeloid lineage cells can be targeted with BP. This has been recently demonstrated in a model of malignant histiocytosis [38]. CLO-containing liposomes were firstly assayed *in vitro* on canine malignant histiocytosis cells, demonstrating a significant inhibition of cell growth. This effect was also found even in nonphagocytic cells, although, for these cells, free CLO was more efficient. *In vivo*, dogs with spontaneous malignant histiocytosis and treated with CLO-containing liposomes elicited significant tumor regression in two of five treated animals. The authors also reported an antitumor activity following i.v. administration of CLO-containing liposomes in several different nonhistiocytic mouse tumor models, thus suggesting the antitumor activity may have been mediated by a combination of both direct and indirect tumor effects [38].

Liposomes have been used to deliver BPs directly to cancer cells (Table 1). Neridronate (NER) encapsulated into liposomes increased the inhibition activity on cell growth on human breast cancer cells (MDA-MB-231) by 50 times, compared to the free drug [39].

Moreover, even at a lower concentration, liposomal NER showed a suppressive effect on tumor cell mobility *in vitro*, whereas free NER showed almost no effect. Reasonably, liposomes should mediate the enhanced bisphosphonate uptake into the cells, although this hypothesis was demonstrated only by indirect evidence by co-encapsulation of fluorescent dye together with the drug. 

In order to directly deliver BP in tumor cells, accumulation in MPS should be avoided. Thus, nanocarriers with stealth properties able to avoid opsonization should be preferred. In the light of this consideration, stealth liposomes encapsulating ZOL (lipoZOL) designed for tumor targeting were developed [40, 42]. ZOL was encapsulated into liposomes by different strategies, and the reverse-phase evaporation technique was selected to achieve the highest encapsulation efficiency (unpublished data). With this technique, the use of an alkaline buffer improved the ZOL solubility into the aqueous phase of liposomes, thus increased the drug encapsulation efficiency up to about 5% [40]. Liposomes were able to significantly prolong ZOL circulation time. Free ZOL was quickly cleared from blood, with 0.1-0.2% of the injected dose still present 1 h after injection. ZOL encapsulation into liposomes, especially PEGylated liposomes, significantly increased ZOL circulation time, with more than 10% of the injected dose still present into the blood 24 h following the injection [42]. Concerning the *in vitro* activity of lipoZOL, contrasting results have been found. In particular, our group demonstrated that the use of lipoZOL, compared with free ZOL, increased the cytotoxic effect until a potentiation factor of about 20 [40]. The effect was confirmed in cell lines of different cancer, namely, prostate, breast, head/neck, lung and pancreas, and multiple myeloma, with an IC50 ranging from 4 to about 200 *μ*M. These data are in contrast with those reported by other authors who found that stealth liposomes containing ZOL did not elicited any significant inhibitory effect on cell from 0.01 to 200 *μ*M [42]. Significant cytotoxicity was found only by using folate-conjugated lipoZOL, especially in cell overexpressing the folate receptor. The discrepancy among the two studies could be ascribed to the different formulations used as well as to the different cell lines.

The *in vivo* antitumor activity of lipoZOL was demonstrated in two different model of tumors, namely, prostate cancer and multiple myeloma [40, 41]. In these experiments, mice treated with lipoZOL, compared to animal with free ZOL, showed a higher tumor weight inhibition and tumor growth delay, together with increased mice survival. As in the case of non-stealth nanocarriers, also stealth liposomes allowed to obtain reduced number of TAM as well as inhibition of the neoangiogenesis [40, 41]. Moreover, no significant changes were found in serum creatinine, urea, and calcium in animals treated with lipoZOL, suggesting the absence of potential adverse effects [40]. In order to overcome technological limits of the lipoZOL, such as low encapsulation efficiency and stability issue of the liposomal formulation, our group recently developed a new nanovector to deliver ZOL in extraskeletal tumor. The new system consists of self-assembling NPs encapsulating ZOL and designed to be prepared before use, thus avoiding storage issues [43, 102]. In particular, the formulation can be prepared by mixing two components, namely, an aqueous solution of ZOL, Ca^2+^/PO_4_^3−^ NPs, and cationic PEGylated liposomes. Ca^2+^/PO_4_^3−^ have already been used to deliver other negatively charged molecules, such as nucleic acids [103]. In the case of BPs, an encapsulation process driven by ionic interactions allowed to overcome the loading issues observed with liposomes. Indeed, in the case of self-assembling NPs, a ZOL encapsulation efficiency 12-fold greater, compared with that obtained with ZOL-containing liposomes, was achieved. The self-assembling NPs increased the growth inhibition of ZOL on different cancer cell lines, compared to free ZOL. The highest cell growth inhibition was observed on breast cancer cells. The anticancer activity of this formulation was also demonstrated *in vivo* in an animal model of prostate cancer. ZOL encapsulated into self-assembling NPs elicited a marked antitumor activity, while free ZOL did not show a significant reduction of tumor growth [43]. The *in vivo* anticancer activities of two different ZOL-containing nanocarriers, namely, lipoZOL and self-assembling NPs, were compared [41]. In this study, self-assembling NPs encapsulating ZOL induced the complete remission of tumour xenografts and an increase of survival time higher than that observed with lipoZOL. This effect was paralleled by a significant increase of both necrotic and apoptotic indexes. NPs, more than lipoZOL, also caused a statistically significant reduction of TAM and displayed a higher neoangiogenesis inhibition. With both nanovectors, toxic effects affecting the mice weight or inducing deaths were not found. Finally, the histological examination of some vital organs such as liver, kidney, and spleen did not find any changes in terms of necrotic effects or modifications in the inflammatory infiltrate [41].

The ability of BPs to bind metal ions was used to prepare BP-complexing superparamagnetic iron oxide nanocrystals with theranostic purposes [44–46]. In a first study, a 5-hydroxy-5, 5-bis(phosphono) pentanoic acid was used, while in the following works more powerful BPs, such as ALE and ZOL, were used. Amino fluorescein or rhodamine were covalently coupled with the nanocrystal, thus allowing to visualize an efficient uptake of the nanovector into two different cell lines [44, 104]. However, cell viability assays demonstrated that ZOL alone had an IC50 at 48 h that was 1 order of magnitude lower than with *γ*Fe_2_O_3_-ZOL nanocrystals. According to the authors, cell proliferation decreases to 75% under an applied magnetic field, compared to 40% without magnetic field [45]. *γ*Fe_2_O_3_-ALE NPs were investigated on different cell lines; however, a clear advantage of the NPs was found only on breast cancer cell [104]. These NPs were also investigated *in vivo* in an experimental model of breast cancer [104]. In this study, tumour growth in animals treated with free ALE and *γ*Fe_2_O_3_-ALE NPs was not significantly different than in control group. NPs used in combination with a magnetic field significantly inhibited tumour growth by about 60% after 5 weeks, with all mice treated that were alive 5 weeks after treatment and did not present significant loss of body weight. However, the lack of control experiments with *γ*Fe_2_O_3_ NPs (NPs without ALE) hampers to affirm that ALE could be responsible for the antitumor affect, while the physical effect of NPs under the magnetic field could be the main responsible of anticancer effect described by the authors.

## 8. Nanotechnology and BPs: Targeting of Bone Tumors

Bone metastasis, especially originating by breast and prostate cancer, are the most frequent form of skeletal neoplasia. In the majority of patients, treatments of bone metastasis are palliative, being aimed to relieve pain, improve function, and prevent complications such as spinal cord compression and pathological fracture. The development of anticancer therapies with high affinity for bone and reduced distribution to other sites is certainly attractive. To this aim, nanovectors targeting hydroxyapatite have been proposed. Hydroxyapatite (Ca_10_(PO_4_)_6_(OH)_2_) is the major inorganic mineral phase present in bone and teeth and not found in other tissues under normal circumstances. Thus, the use of nanocarriers conjugated to BPs that are characterized by high affinity for hydroxyapatite have been proposed. 

A novel amphipathic molecule bearing a bisphosphonate head group,  4-N-(3,5-ditetradecyloxybenzoyl)-aminobutane-1-hydroxy-1,1-bisphosphonic acid disodium salt (BPA), was synthesized and used at different concentrations to prepare liposomes [47]. The presence of the bisphosphonates on the liposome surface was suggested by a zeta potential that was as negative as high the amount of the BPA used in the preparation. BPA-containing liposomes bound hydroxyapatite *in vitro*, depending on the BPA concentration into the carrier, while no binding was found in the case of liposomes prepared without BPA. *In vitro* studies on human osteosarcoma cell line associated to hydroxyapatite demonstrated an increased cytotoxicity of BPA-containing liposomes encapsulating doxorubicin, compared to liposome not containing BPA, this effect being dependant on the amount of BPA used in the preparation [47]. Liposomes containing doxorubicin (DOX) were also conjugated to CLO to target osteosarcoma [105]. DOX-encapsulating BP-conjugated liposomes showed similar antitumor effect on two different osteosarcoma cell lines, compared to DOX in free form or encapsulated into PEGylated liposomes. Moreover, in an experimental model of osteosarcoma, a higher inhibition rate of tumor growth, together with a prolonged survival, was observed when comparing mice treated with DOX-encapsulating BP-conjugated liposomes with the other groups. 

ALE has also been coupled to poly(lactide-co-glycolide) (PLGA) NPs encapsulating doxorubicin [48]. These NPs were investigated in a panel of human cell lines, representative of primary and metastatic bone tumors on which doxorubicin, as free or encapsulated in ALE-conjugated NPs, induced a concentration-dependent inhibition of cell proliferation. *In vivo* studies on an orthotopic mouse model of breast cancer bone metastases demonstrated a reduced incidence of metastases in the case of mice treated with doxorubicin, as free or encapsulated in ALE-conjugated NPs. However, in the case of ALE-conjugated NPs, independently on the presence of doxorubicin, a significant reduction of the osteoclast number was found at the tumor site, reasonably attributed to the ALE activity [48]. PLGA NPs conjugated with ZOL have been recently developed to deliver docetaxel (DCX) to bone [49]. ZOL was conjugated to PLGA-PEG-NH2 and the resulting PLGA-PEG-ZOL was used to prepare the NPs. *In vitro* bone binding affinity showed that PLGA-PEG-ZOL NPs have affinity with human bone powder comparable to that observed for ZOL in solution. On two different breast cancer cell lines, PLGA-PEG-ZOL NPs exhibited significantly higher cytotoxicity compared to DCX, DCX associated to ZOL, and unconjugated NPs at all drug concentrations and different time points. Interestingly, the authors demonstrated that the presence of ZOL on the NP surface affected the pathway for the intracellular uptake. In particular, PEGylated PLGA NPs predominantly followed lysosome through early endosomes which displayed significant colocalization of NPs and lysosomes. On the other hand, ZOL-modified NPs were endocytosed by both clathrin-mediated and caveolae-mediated endocytosis mechanism, where caveolae pathway followed a non-lysosomal route. The different intracellular trafficking of ZOL-coupled and ZOL-free NPs was also confirmed by the prolonged time needed for the exocytosis [49]. Finally, ZOL-coupled NPs showed an enhanced cytotoxic effect that has been attributed to the higher uptake via ZOL-mediated endocytosis. Finally, ALE was also conjugated to a poly(ethylene glycol) (PEG) dendrimer, in combination with paclitaxel to target bone tumors [50]. The pharmacological activity of paclitaxel, in terms of inhibition of cell growth and cell migration, was not altered by conjugation with PEG dendrimer. Moreover, *in vivo* half-life of paclitaxel was significantly improved when administering the conjugate ALE-dendrimer-paclitaxel, compared with free paclitaxel. 

## 9. Concluding Remarks

*In vitro* results have clearly demonstrated that BPs, in addition to inhibiting osteoclast-mediated bone resorption, can exert marked proapoptotic and antiproliferative effects on tumor cells, especially when combined with other standard antineoplastic therapy. *In vivo*, this antitumor effect appears to be better experienced in tumor cells of bone metastases, at least in the majority of experiments performed to date. This may be explained by the high local concentration of BPs in bone relative to the much lower one in other organs and plasma; this feature makes bisphosphonates the drugs of choice in the treatment of bone problems associated with malignancy. However, large-scale clinical trials have investigated the benefit of bisphosphonate therapy in reducing the incidence of SRE in myeloma, in breast cancer metastases, in metastatic prostate cancer, in lung cancer, in renal cell carcinoma, and in other solid tumors. Many *in vivo* tumor models have demonstrated ZOL, PAM, CLO, and IBA antitumor efficacy compared with control.

The use of nanotechnology can open new therapeutic scenario for BPs. Nanocarriers such as conventional liposomes allow to use the BP as potent agent for macrophage depletion. Preferential accumulation of BP in extraskeletal tissue can be achieved by using long circulating nanocarriers, such as lipoZOL and self-assembling NPs. The functionalization of these NPs with ligand, that is, folate or transferrin, able to target cancer cells, can be used to enhance the antitumor activity and to increase the selectivity of the treatment. BP can be conjugated on the surface of nanocarriers, that is, PEGylated PLGA NPs or PEG dendrimer conjugated with the anticancer agent, to be used as targeting moieties, for the treatment of bone cancers. 

Taking together all the scientific papers cited in this paper, the role of BPs in therapy appears underestimated. This class of molecules, especially the third-generation N-BPs as ZOL, can certainly represent a new weapon against cancer, although today they are approved only as antiresorption agent. Of course, new therapeutic indications cannot leave aside the design of a specific delivery system able to change biopharmaceutical characteristics of BPs. In line with this, nanotechnology can certainly represent an attractive opportunity. 

## 10. Future Perspectives

Several strategies could be developed in the next future: the rational use of N-BPs in combination with other target-based agents to overcome escape mechanism occurring in cancer cells; the sequential combination of N-BPs with conventional cytotoxic agents to strengthen their apoptotic and antiangiogenic potential; the administration of N-BPs in metronomic-like modality (low doses for protracted time); the discovery and the targeting of new intracellular molecules found through the use of new advanced molecular technologies, such as DNA microarray. In all these possible perspectives nanotechnology will represent a valid support, also contributing to make these molecules more specific, thus reducing contraindications, for example, osteonecrosis of the jaw, due to the excessive N-BP accumulation in sites where their action is not required. Studies in progress in our labs suggest future applications of BPs also in form of cancer hard to kill, like glioma, and for other applications in the central nervous system, like the treatment of neuropathic pain (data submitted for publication).

## Figures and Tables

**Table 1:** Summary of the most meaningful studies published on nanotechnology to deliver BPs in cancer.

Delivery system	Strategy	Bisphosphonate	Main findings	References
Liposomes	Macrophage depletion	Clodronate	Macrophage elimination in the spleen and liver following i.v. administration.	[21–25]
Liposomes	Macrophage depletion	Clodronate, pamidronate, etidronate	Macrophage elimination in the bloodstream following i.v. administration.	[26]
Liposomes	Macrophage depletion	Clodronate, pamidronate, etidronate	BPs were found to be even 1000 times less active, compared with the corresponding liposome-based formulations; high calcium extracellular concentration resulted in a stronger macrophage depletion; negatively charged unilamellar liposomes favour macrophage depletion.	[23, 24, 27]
Liposomes	Macrophage depletion	Clodronate	Macrophage elimination in draining lymph nodes following subcutaneous footpad administration.	[28]
Liposomes	Macrophage depletion	Clodronate	Intratracheal administration exclusively eliminates macrophages from lung tissues.	[29]
Liposomes	Macrophage depletion	Clodronate	Enhanced tumor growth in an experimental model of liver metastasis.	[30]
Liposomes	Macrophage depletion	Clodronate	Inhibition of the tumor growth in different experimental animal models of cancer; reduction of the blood vessel density in the tumor tissue; reduction of the tumor-associated macrophages and tumor-associated dendritic cells.	[31–33]
Liposomes	Macrophage depletion	Clodronate in combination with sorafenib	Significant inhibition of tumor growth and lung metastasis; reduced tumor angiogenesis.	[34]
Liposomes	Macrophage depletion	Clodronate as adjuvant agent in radiotherapy	Adjuvant agent in the cancer radiotherapy with delayed tumor regrowth.	[35, 36]
Liposomes	Macrophage depletion	Clodronate	Reduced metastasis of human prostate cancer in bone.	[37]
Liposomes	Inhibitory effect on cancer cells	Clodronate	Significant tumor regression.	[38]
Liposomes	Inhibitory effect on cancer cells	Neridronate	Inhibition of cell growth.	[39]
PEGylated liposomes	Targeting of extraskeletal tumors	Zoledronate	Enhanced cytotoxic effect *in vitro*; enhanced inhibition of tumor growth (prostate cancer and multiple myeloma).	[40, 41]
Folate-coupled PEGylated liposomes	Targeting of extraskeletal tumors	Zoledronate	Enhanced cytotoxic effect *in vitro*.	[42]
Self-assembling NPs	Targeting of extraskeletal tumors	Zoledronate	Enhanced cytotoxic effect *in vitro*; enhanced inhibition of tumor growth (prostate cancer).	[41, 43]
Superparamagnetic iron oxide nanocrystals	Theranostic purposes	Alendronate, zoledronate	Decrease cell proliferation *in vivo* and inhibition of tumour growth *in vivo*, only in combination with a magnetic field.	[44–46]
Liposomes	Targeting of doxorubicin to bone tumors	Bisphosphonate head group in a novel amphipathic molecule	Increased cytotoxicity *in vitro* on human osteosarcoma cell line associated to hydroxyapatite.	[47]
Poly(lactide-co-glycolide) NPs	Targeting of doxorubicin to bone tumors	Alendronate conjugated on the nanocarrier surface	Reduced incidence of metastases associated to a significant reduction of the osteoclast number at the tumor site.	[48]
Poly(lactide-co-glycolide) NPs	Targeting of docetaxel to bone tumors	Zoledronate conjugated on the nanocarrier surface	Enhanced cytotoxic effect *in vitro*.	[49]
Poly(ethylene glycol)-dendrimer	Targeting of paclitaxel to bone tumors	Alendronate conjugated to the nanocarrier	Significant improvement of paclitaxel *in vivo* half-life.	[50]

## References

[1] Ross JR, Saunders Y, Edmonds PM (2004). A systematic review of the role of bisphosphonates in metastatic disease. *Health Technology Assessment*.

[2] Fleisch H, Russell RGG, Bisaz S, Casey PA, Mühlbauer RC (1968). The influence of pyrophosphate analogues (diphosphonates) on the precipitation and dissolution of calcium phosphate in vitro and in vivo. *Calcified Tissue Research*.

[3] Russell RG (2011). Bisphosphonates: the first 40 years. *Bone*.

[4] Widler L, Jahnke W, Green JR (2012). The chemistry of bisphosphonates: from antiscaling agents to clinical therapeutics. *Anticancer Agents in Medicinals Chemistry*.

[5] Russell RG (2007). Bisphosphonates: mode of action and pharmacology. *Pediatrics*.

[6] Dunford JE, Thompson K, Coxon FP (2001). Structure-activity relationships for inhibition of farnesyl diphosphate synthase in vitro and inhibition of bone resorption in vivo by nitrogen-containing bisphosphonates. *Journal of Pharmacology and Experimental Therapeutics*.

[7] Green JR (2003). Antitumor effects of bisphosphonates. *Cancer*.

[8] Ebetino FH, Hogan AM, Sun S (2011). The relationship between the chemistry and biological activity of the bisphosphonate. *Bone*.

[9] Plotkin LI, Weinstein RS, Parfitt AM, Roberson PK, Manolagas SC, Bellido T (1999). Prevention of osteocyte and osteoblast apoptosis by bisphosphonates and calcitonin. *The Journal of Clinical Investigation*.

[10] Rogers MJ, Watts DJ, Russell RGG (1994). Inhibitory effects of bisphosphonates on growth of amoebae of the cellular slime mold Dictyostelium discoideum. *Journal of Bone and Mineral Research*.

[11] Rogers MJ (2004). From molds and macrophages to mevalonate: a decade of progress in understanding the molecular mode of action of bisphosphonates. *Calcified Tissue International*.

[12] Rogers MJ, Brown RJ, Hodkin V, Russell RGG, Watts DJ, Blackburn GM (1996). Bisphosphonates are incorporated into adenine nucleotides by human aminoacyl-tRNA synthetase enzymes. *Biochemical and Biophysical Research Communications*.

[13] Frith JC, Monkkonen J, Auriola S, Monkkonen H, Rogers MJ (2001). The molecular mechanism of action of the antiresorptive and anti-inflammatory drug clodronate: evidence for the formation in vivo of a metabolite that inhibits bone resorption andcauses osteoclast and macrophage apoptosis. *Arthritis & Rheumatism*.

[14] Lehenkari PP, Kellinsalmi M, Näpänkangas JP (2002). Further insight into mechanism of action of clodronate: inhibition of mitochondrial ADP/ATP translocase by a nonhydrolyzable, adenine-containing metabolite. *Molecular Pharmacology*.

[15] Halasy-Nagy JM, Rodan GA, Reszka AA (2001). Inhibition of bone resorption by alendronate and risedronate does not require osteoclast apoptosis. *Bone*.

[16] Roelofs AJ, Thompson K, Gordon S, Rogers MJ (2006). Molecular mechanisms of action of bisphosphonates: current status. *Clinical Cancer Research*.

[17] Berenson JR (2011). Antitumor effects of bisphosphonates: from the laboratory to the clinic. *Current Opinion in Supportive & Palliative Care*.

[18] Carmeliet P, Jain RK (2000). Angiogenesis in cancer and other diseases. *Nature*.

[19] Carmeliet P (2003). Angiogenesis in health and disease. *Nature Medicine*.

[20] Caraglia M, Santini D, Marra M, Vincenzi B, Tonini G, Budillon A (2006). Emerging anti-cancer molecular mechanisms of aminobisphosphonates. *Endocrine-Related Cancer*.

[21] Claassen E, van Rooijen N (1984). The effect of elimination of macrophages on the tissue distribution of liposomes containing [^3^H]methotrexate. *Biochimica et Biophysica Acta*.

[22] van Rooijen N, van Nieuwmegen R (1984). Elimination of phagocytic cells in the spleen after intravenous injection of liposome encapsulated dichloromethylene diphosphonate. An enzyme-histochemical study. *Cell and Tissue Research*.

[23] van Rooijen N (1989). The liposome-mediated macrophage ‘suicide’ technique. *Journal of Immunological Methods*.

[24] Mönkkönen J, Pennanen N, Lapinjoki S, Urtti A (1994). Clodronate (dichloromethylene bisphosphonate) inhibits LPS-stimulated IL-6 and TNF production by RAW 264 cells. *Life Sciences*.

[25] van Rooijen N, Claassen E, Gregoriadis G (1988). In vivo elimination of macrophages in spleen and liver, using liposome encapsulated drugs: methods and applications. *Liposomes as Drug Carriers: Trends and Progress*.

[26] van Rooijen N, van Nieuwmegen R, Kamperdijk EWA (1985). Elimination of phagocytic cells in the spleen after intravenous injection of liposome-encapsulated dichloromethylene diphosphonate. Ultrastructural aspects of elimination of marginal zone macrophages. *Virchows Archiv B*.

[27] Pennanen N, Lapinjoki S, Urtti A, Mönkkönen J (1995). Effect of liposomal and free bisphosphonates on the IL-1*β*, IL-6 and TNF*α* secretion from RAW 264 cells in vitro. *Pharmaceutical Research*.

[28] Delemarre FGA, Kors N, Kraal G, van Rooijen N (1990). Repopulation of macrophages in popliteal lymph nodes of mice after liposome-mediated depletion. *Journal of Leukocyte Biology*.

[29] Thepen T, van Rooijen N, Kraal G (1989). Alveolar macrophage elimination in vivo is associated with an increase in pulmonary immune response in mice. *The Journal of Experimental Medicine*.

[30] Heuff G, Oldenburg HSA, Boutkan H (1993). Enhanced tumour growth in the rat liver after selective elimination of Kupffer cells. *Cancer Immunology and Immunotherapy*.

[31] Zeisberger SM, Odermatt B, Marty C, Zehnder-Fjällman AH, Ballmer-Hofer K, Schwendener RA (2006). Clodronate-liposome-mediated depletion of tumour-associated macrophages: a new and highly effective antiangiogenic therapy approach. *British Journal of Cancer*.

[32] Kimura YN, Watari K, Fotovati A (2007). Inflammatory stimuli from macrophages and cancer cells synergistically promote tumor growth and angiogenesis. *Cancer Science*.

[33] Gazzaniga S, Bravo AI, Guglielmotti A (2007). Targeting tumor-associated macrophages and inhibition of MCP-1 reduce angiogenesis and tumor growth in a human melanoma xenograft. *Journal of Investigative Dermatology*.

[34] Zhang W, Zhu XD, Sun HC (2010). Depletion of tumor-associated macrophages enhances the effect of sorafenib in metastatic liver cancer models by antimetastatic and antiangiogenic effects. *Clinical Cancer Research*.

[35] Sherman ML, Datta R, Hallahan DE, Weichselbaum RR, Kufe DW (1991). Regulation of tumor necrosis factor gene expression by ionizing radiation in human myeloid leukemia cells and peripheral blood monocytes. *The Journal of Clinical Investigation*.

[36] Meng Y, Beckett MA, Liang H (2010). Blockade of tumor necrosis factor *α* signaling in tumor-associated macrophages as a radiosensitizing strategy. *Cancer Research*.

[37] Kim SW, Kim JS, Papadopoulos J (2011). Consistent interactions between tumor cell IL-6 and macrophage TNF-*α* enhance the growth of human prostate cancer cells in the bone of nude mouse. *International Immunopharmacology*.

[38] Hafeman S, London C, Elmslie R, Dow S (2010). Evaluation of liposomal clodronate for treatment of malignant histiocytosis in dogs. *Cancer Immunology and Immunotherapy*.

[39] Chebbi I, Migianu-Griffoni E, Sainte-Catherine O, Lecouvey M, Seksek O (2010). In vitro assessment of liposomal neridronate on MDA-MB-231 human breast cancer cells. *International Journal of Pharmaceutics*.

[40] Marra M, Salzano G, Leonetti C (2011). Nanotechnologies to use bisphosphonates as potent anticancer agents: the effects of zoledronic acid encapsulated into liposomes. *Nanomedicine*.

[41] Marra M, Salzano G, Leonetti C (2012). New self-assembly nanoparticles and stealth liposomes for the delivery of zoledronic acid: a comparative study. *Biotechnology Advances*.

[42] Shmeeda H, Amitay Y, Gorin J (2010). Delivery of zoledronic acid encapsulated in folate-targeted liposome results in potent in vitro cytotoxic activity on tumor cells. *Journal of Controlled Release*.

[43] Salzano G, Marra M, Porru M (2011). Self-assembly nanoparticles for the delivery of bisphosphonates into tumors. *International Journal of Pharmaceutics*.

[44] Lalatonne Y, Paris C, Serfaty JM, Weinmann P, Lecouvey M, Motte L (2008). Bis-phosphonates-ultra small superparamagnetic iron oxide nanoparticles: a platform towards diagnosis and therapy. *Chemical Communications*.

[45] Benyettou F, Lalatonne Y, Sainte-Catherine O, Monteil M, Motte L (2009). Superparamagnetic nanovector with anti-cancer properties: *γ*Fe_2_O_3_@Zoledronate. *International Journal of Pharmaceutics*.

[46] Benyettou F, Guenin E, Lalatonne Y, Motte L (2011). Microwave assisted nanoparticle surface functionalization. *Nanotechnology*.

[47] Anada T, Takeda Y, Honda Y, Sakurai K, Suzuki O (2009). Synthesis of calcium phosphate-binding liposome for drug delivery. *Bioorganic & Medicinal Chemistry Letters*.

[48] Salerno M, Cenni E, Fotia C (2010). Bone-targeted doxorubicin-loaded nanoparticles as a tool for the treatment of skeletal metastases. *Current Cancer Drug Targets*.

[49] Ramanlal Chaudhari K, Kumar A, Megraj Khandelwal VK (2012). Bone metastasis targeting: a novel approach to reach bone using Zoledronate anchored PLGA nanoparticle as carrier system loaded with Docetaxel. *Journal of Controlled Release*.

[50] Clementi C, Miller K, Mero A, Satchi-Fainaro R, Pasut G (2011). Dendritic poly(ethylene glycol) bearing paclitaxel and alendronate for targeting bone neoplasms. *Molecular Pharmaceutics*.

[51] Kavanagh KL, Guo K, Dunford JE (2006). The molecular mechanism of nitrogen-containing bisphosphonates as anti-osteoporosis drugs: crystal structure and inhibition of farnesyl pyrophosphate synthase. *Proceedings of the National Academy of Sciences of the United States of America*.

[52] Thompson K, Rogers MJ, Coxon FP, Crockett JC (2006). Cytosolic entry of bisphosphonate drugs requires acidification of vesicles after fluid-phase endocytosis. *Molecular Pharmacology*.

[53] Ibrahim A, Scher N, Williams G (2003). Approval summary for zoledronic acid for treatment of multiple myeloma and cancer bone metastases. *Clinical Cancer Research*.

[54] Chen T, Berenson J, Vescio R (2002). Pharmacokinetics and pharmacodynamics of zoledronic acid in cancer patients with bone metastases. *Journal of Clinical Pharmacology*.

[55] Skerjanec A, Berenson J, Hsu CH (2003). The pharmacokinetics and pharmacodynamics of zoledronic acid in cancer patients with varying degrees of renal function. *Journal of Clinical Pharmacology*.

[56] Lin JH (1996). Bisphosphonates: a review of their pharmacokinetic properties. *Bone*.

[57] Barrett J, Worth E, Bauss F, Epstein S (2004). Ibandronate: a clinical pharmacological and pharmacokinetic update. *Journal of Clinical Pharmacology*.

[58] Weiss HM, Pfaar U, Schweitzer A, Wiegand H, Skerjanec A, Schran H (2008). Biodistribution and plasma protein binding of zoledronic acid. *Drug Metabolism and Disposition*.

[59] Pickering LM, Mansi JL (2003). Adhesion of breast cancer cells to extracellular matrices is inhibited by zoledronic acid and enhanced by aberrant Ras signaling. *American Society of Clinical Oncology*.

[60] Wood J, Bonjean K, Ruetz S (2002). Novel antiangiogenic effects of the bisphosphonate compound zoledronic acid. *Journal of Pharmacology and Experimental Therapeutics*.

[61] Croucher PI, de Raeve H, Perry MJ (2003). Zoledronic acid treatment of 5T2MM-bearing mice inhibits the development of myeloma bone disease: evidence for decreased osteolysis, tumor burden and angiogenesis, and increased survival. *Journal of Bone and Mineral Research*.

[62] Dieli F, Gebbia N, Poccia F (2003). Induction of *γδ* T-lymphocyte effector functions by bisphosphonate zoledronic acid in cancer patients in vivo. *Blood*.

[63] Santini D, Galluzzo S, Vincenzi B (2007). New developments of aminobisphosphonates: the double face of Janus. *Annals of Oncology*.

[64] Benford HL, Frith JC, Auriola S, Mönkkönen J, Rogers MJ (1999). Farnesol and geranylgeraniol prevent activation of caspases by aminobisphosphonates: biochemical evidence for two distinct pharmacological classes of bisphosphonate drugs. *Molecular Pharmacology*.

[65] Coxon FP, Helfrich MH, van’t Hof R (2000). Protein geranylgeranylation is required for osteoclast formation, function, and survival: inhibition by bisphosphonates and GGTI-298. *Journal of Bone and Mineral Research*.

[66] Boissier S, Ferreras M, Peyruchaud O (2000). Bisphosphonates inhibit breast and prostate carcinoma cell invasion, an early event in the formation of bone metastases. *Cancer Research*.

[67] Misso G, Porru M, Stoppacciaro A (2012). Evaluation of the in vitro and in vivo antiangiogenic effects of denosumab and zoledronic acid. *Cancer Biology and Therapy*.

[68] Bezzi M, Hasmim M, Bieler G, Dormond O, Rüegg C (2003). Zoledronate sensitizes endothelial cells to tumor necrosis factor-induced programmed cell death: evidence for the suppression of sustained activation of focal adhesion kinase and protein kinase B/Akt. *The Journal of Biological Chemistry*.

[69] Marra M, Abbruzzese A, Addeo R (2009). Cutting the limits of aminobisphosphonates: new strategies for the potentiation of their anti-tumour effects. *Current Cancer Drug Targets*.

[70] Caraglia M, D’Alessandro AM, Marra M (2004). The farnesyl transferase inhibitor R115777 (Zarnestra) synergistically enhances growth inhibition and apoptosis induced on epidermoid cancer cells by Zoledronic acid (Zometa) and Pamidronate. *Oncogene*.

[71] Senaratne SG, Mansi JL, Colston KW (2002). The bisphosphonate zoledronic acid impairs Ras membrane [correction of impairs membrane] localisation and induces cytochrome c release in breast cancer cells. *British Journal of Cancer*.

[72] Sewing L, Steinberg F, Schmidt H, Göke R (2008). The bisphosphonate zoledronic acid inhibits the growth of HCT-116 colon carcinoma cells and induces tumor cell apoptosis. *Apoptosis*.

[73] Fujita M, Tohi M, Sawada K (2012). Involvement of the mevalonate pathway in the antiproliferative effect of zoledronate on ACHN renal cell carcinoma cells. *Oncology Reports*.

[74] Ferretti G, Fabi A, Carlini P (2005). Zoledronic-acid-induced circulating level modifications of angiogenic factors, metalloproteinases and proinflammatory cytokines in metastatic breast cancer patients. *Oncology*.

[75] Herbst RS, Khuri FR (2003). Mode of action of docetaxel—a basis for combination with novel anticancer agents. *Cancer Treatment Reviews*.

[76] Ullén A, Lennartsson L, Harmenberg U (2005). Additive/synergistic antitumoral effects on prostate cancer cells in vitro following treatment with a combination of docetaxel and zoledronic acid. *Acta Oncologica*.

[77] Fabbri F, Brigliadori G, Carloni S (2008). Zoledronic acid increases docetaxel cytotoxicity through pMEK and Mcl-1 inhibition in a hormone-sensitive prostate carcinoma cell line. *Journal of Translational Medicine*.

[78] Karabulut B, Erten C, Gul MK (2009). Docetaxel/zoledronic acid combination triggers apoptosis synergistically through downregulating antiapoptotic Bcl-2 protein level in hormone-refractory prostate cancer cells. *Cell Biology International*.

[79] Marra M, Santini D, Meo G (2009). CYR61 downmodulation potentiates the anticancer effects of zoledronic acid in androgen-independent prostate cancer cells. *International Journal of Cancer*.

[80] Koul HK, Koul S, Meacham RB (2012). New role for an established drug? Bisphosphonates as potential anticancer agents. *Prostate Cancer and Prostatic Diseases*.

[81] Corey E, Brown LG, Quinn JE (2003). Zoledronic acid exhibits inhibitory effects on osteoblastic and osteolytic metastases of prostate cancer. *Clinical Cancer Research*.

[82] Croucher PI, de Raeve H, Perry MJ (2003). Zoledronic acid treatment of 5T2MM-bearing mice inhibits the development of myeloma bone disease: evidence for decreased osteolysis, tumor burden and angiogenesis, and increased survival. *Journal of Bone and Mineral Research*.

[83] Alvarez E, Westmore M, Galvin RJS (2003). Properties of bisphosphonates in the 13762 rat mammary carcinoma model of tumor-induced bone resorption. *Clinical Cancer Research*.

[84] Guenther A, Gordon S, Tiemann M (2010). The bisphosphonate zoledronic acid has antimyeloma activity in vivo by inhibition of protein prenylation. *International Journal of Cancer*.

[85] Zheng Y, Zhou H, Brennan K (2007). Inhibition of bone resorption, rather than direct cytotoxicity, mediates the anti-tumour actions of ibandronate and osteoprotegerin in a murine model of breast cancer bone metastasis. *Bone*.

[86] Croucher PI, Shipman CM, van Camp B, Vanderkerken K (2003). Bisphosphonates and osteoprotegerin as inhibitors of myeloma bone disease. *Cancer*.

[87] Cruz JC, Alsina M, Craig F (2001). Ibandronate decreases bone disease development and osteoclast stimulatory activity in an in vivo model of human myeloma. *Experimental Hematology*.

[88] Neudert M, Fischer C, Krempien B, Bauss F, Seibel MJ (2003). Site-specific human breast cancer (MDA-MB-231) metastases in nude rats: model characterisation and in vivo effects of ibandronate on tumour growth. *International Journal of Cancer*.

[89] van der Pluijm G, Que I, Sijmons B (2005). Interference with the microenvironmental support impairs the de novo formation of bone metastases in vivo. *Cancer Research*.

[90] Padalecki SS, Carreon M, Grubbs B, Cui Y, Guise TA (2003). Androgen deprivation therapy enhances bone loss and prostate cancer metastases to bone: prevention by zoledronic acid. *Oncology*.

[91] Lu S, Zhang J, Zhou Z (2008). Synergistic inhibitory activity of zoledronate and paclitaxel on bone metastasis in nude mice. *Oncology Reports*.

[92] Ottewell PD, Deux B, Mönkkönen H (2008). Differential effect of doxorubicin and zoledronic acid on intraosseous versus extraosseous breast tumor growth in vivo. *Clinical Cancer Research*.

[93] Santini D, Vincenzi B, Galluzzo S (2007). Repeated intermittent low-dose therapy with zoledronic acid induces an early, sustained, and long-lasting decrease of peripheral vascular endothelial growth factor levels in cancer patients. *Clinical Cancer Research*.

[94] Auger MJ, Ross JA, Lewis CE, O'Donnell McGee J (1992). The biology of the macrophage. *The Macrophage: The Natural Immune System*.

[95] Speert DP, Lewis CE, O'Donnell McGee J (1992). Macrophages in bacterial infection. *The Macrophage: The Natural Immune System*.

[96] Unanue ER, Allen PM (1987). The basis for the immuno- regulatory role of macrophages and other accessory cells. *Science*.

[97] Fidler IJ (1988). Targeting of immunomodulators to mononuclear phagocytes for therapy of cancer. *Advanced Drug Delivery Reviews*.

[98] Rees RC, Parry H, Lewis CE, O'Donnell McGee J (1992). Macrophages in tumour immunology. *The Macrophage: The Natural Immune System*.

[99] Hao NB, Lü MH, Fan YH (2012). Macrophages in tumor microenvironments and the progression of tumors. *Clinical and Developmental Immunology*.

[100] Moghimi SM, Hunter AC, Andresen TL (2012). Factors controlling nanoparticle pharmacokinetics: an integrated analysis and perspective. *Annual Review of Pharmacological Toxicology*.

[101] Halin S, Rudolfsson SH, van Rooijen N, Bergh A (2009). Extratumoral macrophages promote tumor and vascular growth in an orthotopic rat prostate tumor model. *Neoplasia*.

[102] Salzano G, Marra M, Leonetti C (2011). Nanotechnologies to use zoledronic acid as a potent antitumoral agent. *Journal of Drug Delivery Science and Technology*.

[103] Giger EV, Puigmartí-Luis J, Schlatter R, Castagner B, Dittrich PS, Leroux JC (2011). Gene delivery with bisphosphonate-stabilized calcium phosphate nanoparticles. *Journal of Controlled Release*.

[104] Benyettou F, Lalatonne Y, Chebbi I (2011). A multimodal magnetic resonance imaging nanoplatform for cancer theranostics. *Physical Chemistry Chemical Physics*.

[105] Wu D, Wan M (2012). Methylene diphosphonate-conjugated adriamycin liposomes: preparation, characteristics, and targeted therapy for osteosarcomas in vitro and in vivo. *Biomedical Microdevices*.

